# A Retrospective Cohort Analysis of Limb Salvage Surgery Using Mega Prosthesis in Bone Tumours at a Tertiary Care Centre in Eastern India

**DOI:** 10.7759/cureus.28959

**Published:** 2022-09-08

**Authors:** Nishant Kashyap, Ritesh Runu, Wasim Ahmed, Indrajeet Kumar, Abhijeet Subash

**Affiliations:** 1 Orthopaedics, Indira Gandhi Institute of Medical Sciences, Patna, Patna, IND

**Keywords:** mega prosthesis, orthopaedics, gaint cell tumour, bone tumours, limb salvage surgery

## Abstract

Background

The method known as "limb salvage surgery" (LSS) aids in the removal of extremity tumours, and reconstruction is completed with satisfactory oncologic, functional, and cosmetic outcomes. Oncologic clearance is given first priority, followed by functional outcomes. Worldwide, the trend has already shifted away from amputations and toward limb salvage surgery for eligible patients due to efficient chemotherapy regimens, improved imaging techniques, precise administration of enhanced radiation, better reconstructive choices, and developments in bio-engineering. The purpose of the present study was to determine the clinicopathological characteristics, surgical techniques, functional outcome, and prognostic factors of limb salvage surgery performed using mega prosthesis in primary malignant or benign resectable tumours.

Methods

Our retrospective cohort study was carried out over a period of two years and included 28 patients who received care for bone tumours. The data gathered comprised the demographic profile, clinical characteristics, histological characteristics, treatments given, functional results, and survival. LSS was performed on all patients by orthopaedics oncologists trained in the surgical oncology department. Following surgery, during the first two years, patients were examined at every three-month interval, then every six months until the fifth year, and then once a year after that. The Kaplan-Meier method was utilized to determine the median follow-up and recurrence-free survival (RFS).

Results

In our study, the mean age of study subjects was 30.0±10.9 years. Almost all of the subjects included in the study had lower limb bone tumours (96.4%). The most common site for the tumour was the distal femur (57.1%) followed by the proximal femur (32.2%). The most common type of benign tumour was giant cell tumour (GCT) (53.6%), including recurrences of giant cell tumour (GCT), and among malignant tumours, osteosarcoma was the most common (25.0%). The mean surgical resection of bone in limb salvage surgery was 125.2±24.2 mm. The most common post-operative complication was leg length discrepancy (LLD) among 25.0% of subjects, which was managed by shoe raise. The overall mean musculoskeletal tumour society (MTSS) score after LSS was 25.0±4.3. Using the Kaplan-Meier method analysis, we found that relapse-free survival was 83.7% among enrolled subjects at a median follow-up period of 80 months.

Conclusion

It can be difficult to surgically treat patients who have malignant bone tumours. In limb-sparing surgery for bone tumours, the modular segmental-replacement system prosthesis that we preferred produced satisfactory results in terms of tumour control and limb function. To get good long-term results, the case selection must be appropriate.

## Introduction

Amputation had previously been the norm for the majority of sarcoma cases, but limb-sparing surgery for most malignant bone tumours emerged in the 1980s. For over 90% of patients with limb malignant bone tumours, limb-sparing surgery is now regarded as safe and common [1]. One possibility for the reconstruction of limb-salvage surgery in these bone malignancies is a mega prosthesis [2]. Due to their availability, ease of use, immediate fixation, early weight bearing, relatively quick recovery of function, excellent cosmetic appearance, and 70% survival rate, modular mega prostheses are now the most common approach for reconstruction after segmental resection of the long bones in the extremities [3].

Mega prostheses, however, are still unable to address cases of bone tumours and achieve results on par with primary joint replacement. The primary issue is to reduce the rate of non-mechanical consequences associated with surgery, such as the risk of wound necrosis and dehiscence, deep infection, and local tumour recurrence [4,5]. The purpose of this study was to determine the clinicopathological characteristics, surgical techniques, functional outcome, and prognostic factors of limb salvage surgery (LSS) performed using mega prosthesis in primary malignant or benign resectable tumours.

## Materials and methods

After receiving approval from the Institutional Review Board (IRB), our study was carried out in the department of orthopaedics at the Indira Gandhi Institute of Medical Sciences (IGIMS) in Patna (IRB approval letter number: 32/IEC/IGIMS/2020). A retrospective cohort analysis was carried out over a period of two years from February 2020 to January 2022. Patients diagnosed with bone tumours and treated for them between January 2015 and January 2022 were included in the study. For the purpose of collecting data, patient files, the surgical register, histopathological reports (HPRs), treatment diaries, and follow-up information were analysed. The gathered data was entered into an MS Excel (Microsoft Corporation, 2018) spreadsheet under the headings: the demographic profile, clinical characteristics, histological characteristics, treatments given, functional results, and survival.

To confirm the histological diagnosis, including the grading of the tumour (I, II A, II B, and III), a Jamshidi needle (J needle, SureTech Medical Inc., Mumbai, India) biopsy was performed. When J needle biopsy was unsuccessful after two attempts or when there was a difference between the radiological and histological findings, an open biopsy with a precisely planned longitudinal incision was carried out. Digital x-ray and magnetic resonance imaging (MRI) of the affected area, a chest computed tomography (CT) scan, and a bone scan were all part of the first workup. During the multidisciplinary tumour board, all cases were discussed.

LSS was performed on all patients by orthopaedics oncologists trained in the surgical oncology department. For reconstruction, we employed a custom-made prosthesis. The plain x-ray scanogram was used to take measurements for a custom prosthesis. Adjuvant therapy was withheld until wound healing if there was associated surgical morbidity such as surgical site infection or delayed wound healing.

Following treatment, during the first two years, patients were examined at every three-month interval, then every six months until the fifth year, and then once a year. Physical examinations and x-rays (local parts and chest) were taken at each follow-up session. Whenever concerning signs were seen on chest x-rays, a CT scan of the chest was performed both annually and as needed. Relapses of disease, whether local or systemic, are defined as events.

For the functional assessment [6], the musculoskeletal tumour society (MTSS) score system was utilised. An improved functional outcome is indicated by a higher score. The physiotherapist computed the score, and data were collected prospectively and saved in the record.

Statistical analysis

MS Excel was used to enter the data that had been obtained. The analysis software used was IBM Statistical Package for the Social Sciences (SPSS) for Windows, version 20.0 (IBM Corp., Armonk, New York, USA). Data analysis methods included descriptive and inferential statistics. The Kaplan-Meier (KM) method was utilized to determine the median follow-up and recurrence-free survival (RFS). RFS was defined as the period of time between treatment completion and the earliest of the following three events: death, disease relapse, or last follow-up. The statistical test's level of significance was set at 5%.

## Results

In our study, the mean age of study subjects was 30.0±10.9 years and ranged between 18 years and 60 years. Around three-fourths of subjects with bone tumours were males (78.6%). Almost all of the subjects included in the study had lower limb bone tumours (96.4%). Half of the tumours (50.0%) were located on the left-side limbs and the remaining half (50.0%) were located on the right-side limbs. The most common site for the tumours was the distal femur (57.1%) followed by the proximal femur (32.2%). In 7.1% of subjects, the proximal humerus bone was having tumour (Table 1).

**Table 1:** Baseline characteristics of study subjects (N=28). #Mean±SD (range).

Variables	Number	%
Age (in years)	30.0±10.9 (18-60) ^#^	
Gender		
Male	22	78.6
Female	6	21.4
Limb involvement		
Lower	27	96.4
Upper	1	3.6
Side of the involved limb		
Left	14	50.0
Right	14	50.0
Bone part		
Proximal femur	9	32.2
Distal femur	16	57.1
Proximal humerus	2	7.1
Proximal tibia	1	3.6

The histopathological examination showed that 71.4% of subjects had benign tumours and the remaining 28.6% of subjects had malignant tumours. The most common type of benign tumour was giant cell tumour (GCT), including recurrence of GCT (53.6%) and among malignant tumours, osteosarcoma was the most common one (25.0%). The histopathological grading of the tumour showed that more than half of tumours (53.6%) were falling under grade II B and only 3.6% of tumours were falling under grade III (Table 2).

**Table 2:** Histopathology of tumours among study subjects (N=28).

Histopathology	Number	%
Nature of tumour		
Benign	20	71.4
Malignant	8	28.6
Type of tumour		
Chondromyxoid fibroma	1	3.6
Giant cell tumour	15	53.6
Multiple myeloma	1	3.6
Osteosarcoma	7	25.0
Recurrent giant cell tumour	4	14.2
Grading of tumour		
II A	15	53.6
II B	12	42.8
III	1	3.6

**Figure 1:**
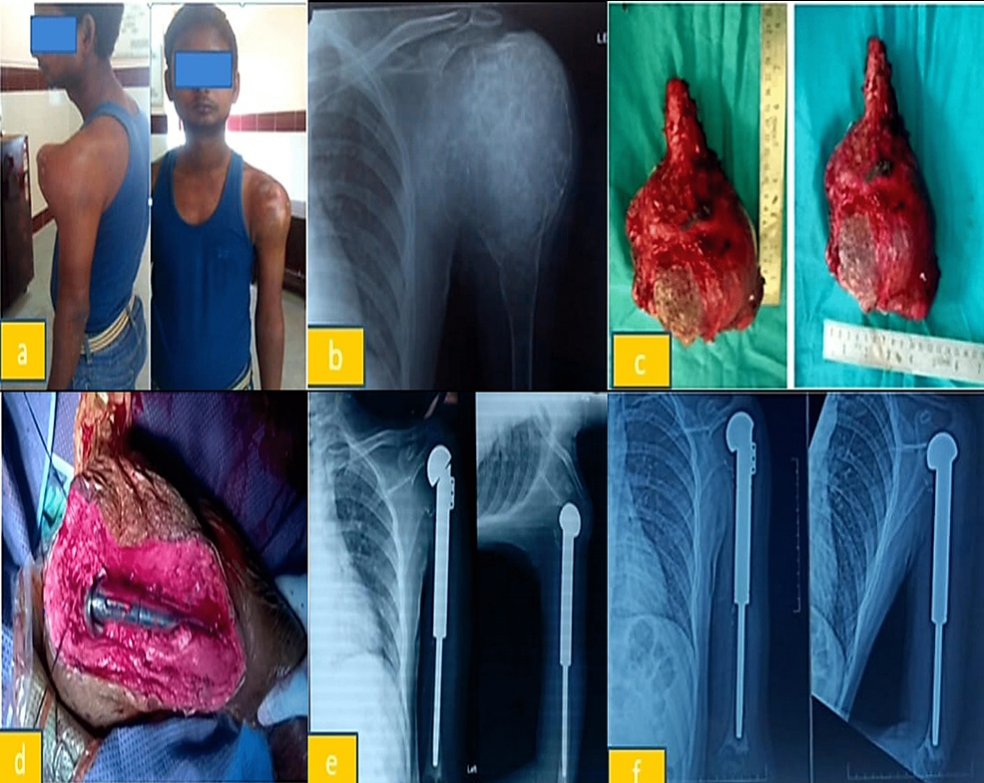
Chondromyxoid fibroma of the left proximal humerus. (a) Pre-operative photograph of a patient with chondromyxoid fibroma of the left proximal humerus. (b) Pre-operative x-ray of the left proximal humerus. (c) Excised left proximal humerus tumour. (d) Intraoperative photograph of the implanted left proximal humerus mega prosthesis. (e) Three-month post-operative x-ray. (f) Six-year x-ray follow-up.

Figure 1 shows the patient with a chondromyxoid fibroma of the left proximal humerus with an excised left proximal humerus tumour and an implanted left proximal humerus mega prosthesis.

The radiological examination showed that in only one subject the tumour had metastasised (3.6%). Among enrolled subjects, 39.3% of subjects were already on chemotherapy prior to limb salvage surgery and 35.7% of subjects were on chemotherapy after limb salvage surgery. The mean surgical resection of bone in limb salvage surgery was 125.2±24.2 mm, and it ranged between 35 mm and 155 mm (Table 3).

**Table 3:** Treatment of tumours among study subjects (N=28). #Mean±SD (range).

Variables	Number	%
Metastasis		
Yes	1	3.6
No	27	96.4
Chemotherapy		
Pre-treatment	11	39.3
Post-treatment	10	35.7
Surgical approach		
Anterior midline	1	3.6
Anterior midline distal thigh and knee	6	21.4
Anterior midline knee and proximal leg	2	7.1
Deltoid-pectoral approach	1	3.6
Lateral distal thigh	6	21.4
Lateral proximal thigh and hip	8	28.6
Medial distal thigh	4	14.3
Treatment		
Hinge knee distal femur mega prosthesis	16	57.1
Long stem modular bipolar prosthesis hip	1	3.6
Proximal femur mega prosthesis	8	28.6
Proximal humerus mega prosthesis	1	3.6
Proximal tibia hinge knee prosthesis	2	7.1
Surgical resection (in mm)	125.2±24.2 (35-155) ^#^	

The most common post-operative complications included delayed wound healing (7.1%), which was managed by prolonged dressing then healed with a scar; extension lag ≥ 30 degrees (10.7%), which was managed conservatively; leg length discrepancy (LLD) among 25.0% of subjects (LLD: 2 cm among 14.3% of subjects and LLD: 3 cm among 10.7% of subjects), which was managed by shoe raise (Table 4).

**Table 4:** Intra-op and early post-operative complications following limb salvage surgery among study subjects (n=28). *Multiple responses.

Complications*	Number (%)	Treatment
Knee extensor weakness	1 (3.6)	Conservative
Complete claw left hand	1 (3.6)	Conservative
Delayed wound healing	2 (7.1)	Dressing till healing completed
Extension lag ≥30 degree	3 (10.7)	Conservative
Intra-op popliteal artery injury	2 (7.1)	Repair popliteal artery
Leg length discrepancy	7 (25.0)	Shoes raise
Wasting of glutaeal and thigh muscles	1 (3.6)	Conservative
Nil	13 (46.5)	Nil

**Figure 2:**
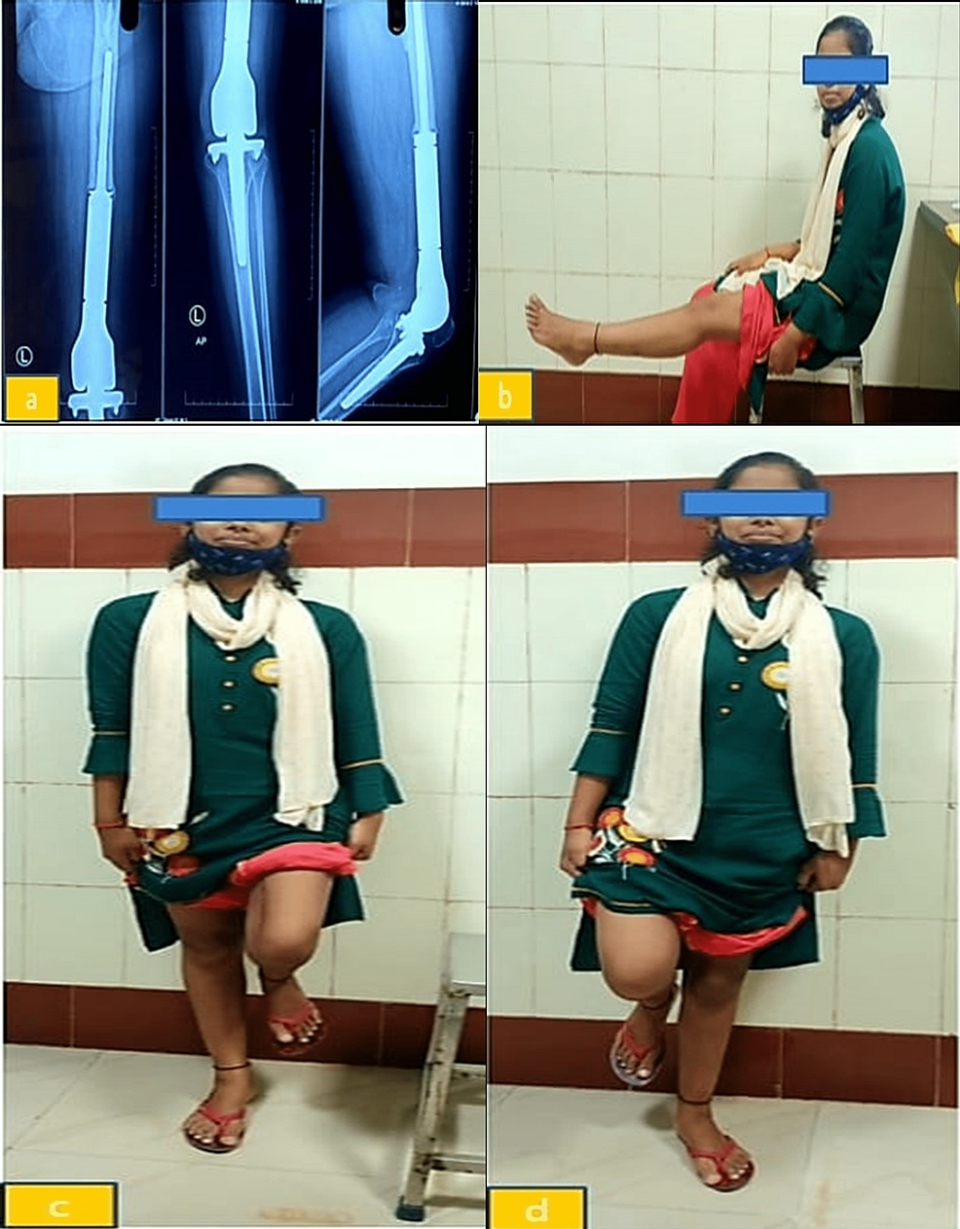
Outcome of limb salvage surgery for osteosarcoma of the distal femur with hinge knee mega prosthesis. (a) X-ray of the left limb following limb salvage surgery for osteosarcoma of the distal femur with hinge knee mega prosthesis at two-year follow-op. (b) Full extension of knee in the left prosthetic limb. (c) Standing on the normal right leg with 90-degree flexion in the left prosthetic limb. (d) Standing on a prosthetic left limb with 90-degree flexion in the normal right leg.

In our study, Figure 2 shows that there was full extension of the knee in the left prosthetic limb, there was 90-degree flexion in the left prosthetic limb while standing on the normal right leg, and there was 90-degree flexion in the normal right leg while standing on a prosthetic left limb following limb salvage surgery for osteosarcoma distal femur with hinge knee mega prosthesis.

The overall mean MTSS score after LSS was 25.0±4.3 ranging between 12 and 29. The mean MTSS score after LSS for proximal femur tumour was 25.0±4.8, for distal femur tumour, the mean MTSS score after LSS was 26.6±1.4 and for proximal tibia tumour, the mean MTSS score after LSS was 16.5±6.4. During follow-up, local recurrence along with inguinal lymph node metastasis was observed among 7.1% of subjects, and multiple vertebral metastases were observed among 3.6% of subjects (Table 5).

**Table 5:** Functional outcome using musculoskeletal tumour society scores (MTSS) among study subjects (N=28). *Multiple responses, #Mean±SD (range).

MTSS score	Score^#^	Percentage^#^
Overall	25.0±4.3 (12-29)	83.4±14.2 (40-97)
Proximal femur tumour	25.0±4.8 (14-29)	83.3±16.1 (47-97)
Distal femur tumour	26.6±1.4 (25-28)	88.6±4.6 (83-93)
Proximal tibia tumour	16.5±6.4 (12-21)	55.0±21.2 (40-70)
Pattern of recurrence*	Number	%
Local recurrence	2	7.1
Inguinal lymph node metastasis	2	7.1
Multiple vertebral metastasis	1	3.6
Nil	21	89.3

**Figure 3:**
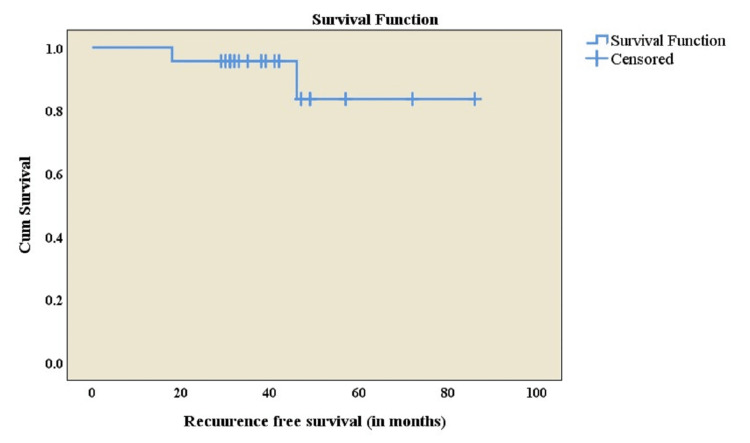
Kaplan-Meir curve for relapse‑free survival study subjects who received limb salvage surgery (N=28).

As this retrospective prospective cohort study shows preliminary results, and not all enrolled subjects have completed the five years of follow-up period including no deaths, we have only done recurrence-free survival using the Kaplan-Meier method for the median follow-up period (80 months) and found that relapse-free survival was 83.7% among enrolled subjects (Figure 3).

**Figure 4:**
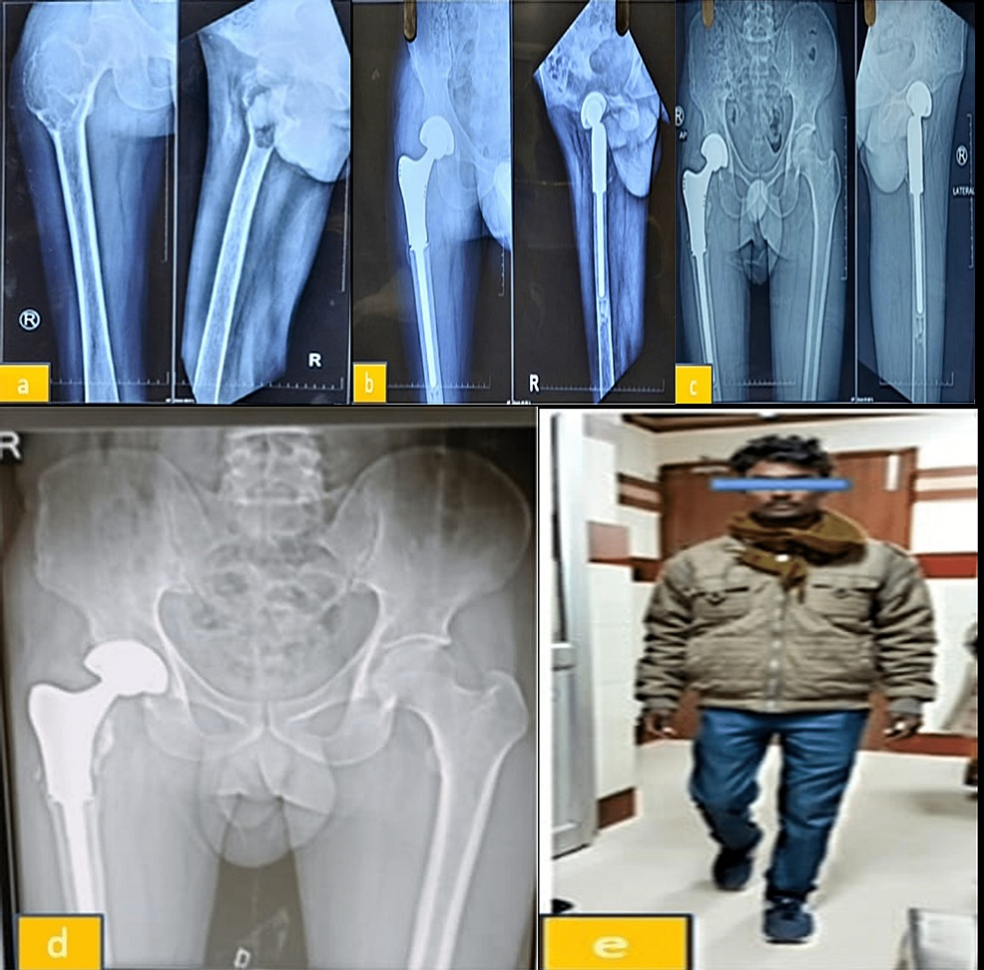
The bone growth around the mega prosthesis. (a) X-ray of the right proximal femur GCT (Enneking's stage IIA). (b) X-ray of the right proximal femur at three-month follow-up. (c) X-ray of the right proximal femur at 2.5-year follow-up. (d) X-ray of the right proximal femur at three-year follow-up showing new bone formation at the calcar of the endoprosthesis. (e) Patient photograph while walking at three-year follow-up. GCT: giant cell tumour.

One more interesting finding observed in our study that there was bone growth or deposition on the mega prosthesis in three subjects (10.7%), which in turn will minimise the chances of loosening of the graft or dislocation of the graft or periprosthetic fracture (Figure 4).

## Discussion

Bone tumours that are primary malignant are very infrequent lesions. Trans bone amputations or disarticulations were the standard of care before the 1970s, with appalling survival rates (10% to 20%). Survival rates increased in the 1970s and 1980s as more potent chemotherapeutic drugs and treatment methods were introduced. This, together with improved imaging techniques and implants, allows for a change in management strategy for limb preservation [7-9].

The most prevalent malignant bone tumour is osteosarcoma [10]. In our analysis, it represented 25% of all bone cancers. Prior to the advent of chemotherapy in the 1970s, surgical excision (usually amputation) was the mainstay of osteosarcoma treatment. The majority of patients eventually acquired recurrent disease, which often manifested as lung metastases, despite such strong local control in more than 80% of cases [10]. The high probability of recurrence suggests that most patients already have the micro-metastatic disease when they receive their initial diagnosis.

Theoretically, "neoadjuvant (pre-operative) chemotherapy" has a benefit in that it can treat these occult micrometastases. It has been shown that shrinking the tumour and "sterilising" the reactive zone around the tumour by eliminating microscopic disease at the primary lesion's periphery, it can facilitate eventual surgical excision. Additionally, certain patients with a relative contraindication to limb salvage, such as an upper extremity pathologic fracture, may be able to consider it if chemotherapy is used and has a positive response [11,12].

The prognosis is better for patients whose tumours histopathology have responded favourably to neoadjuvant chemotherapy (>95% tumour cell death or necrosis) than for patients whose tumours have not [13]. Therefore, neoadjuvant chemotherapy is a standard treatment for osteosarcoma and Ewing's sarcoma. In our study, three out of 28 individuals had tumour relapses during the follow-up period. The projected relapse-free survival rate for 80 months was 83.7%. Similar studies in other countries reveal five-year survival rates ranging from 28% to 76% [14]. Since benign tumours made up the majority of the study's cases, relapse-free survival rates may have been greater. It also gives critical risk information to oncologists.

Nowadays, wide resection and limb preservation with or without reconstruction are safe treatments for 80% to 85% of patients with primary malignant bone tumours involving the extremities, such as osteosarcoma, Ewing's sarcoma, and chondrosarcoma. In the treatment of individuals with high-grade osteosarcoma, limb-salvage surgery was as safe as an amputation. After that, numerous recent studies have shown that limb preservation is typical in bone tumours [15-17].

Arthrodesis and arthroplasty are the two categories under which limb-salvage procedures fall. The joint is preserved through arthroplasty. A metallic prosthesis or an allograft can be used to achieve this. Due to the fact that early metal designs were custom-made, there were noticeable manufacturing delays between diagnosis and reconstruction, which reduced intraoperative flexibility. Malignant bone tumours, on the other hand, are dynamic tumours that transform as time and treatment progress.

As a result, modular prosthetics are presently used in the endoprosthetic reconstruction. Based on the overall amount of tissue removed, intraoperative flexibility is made possible by the prosthetic design's modularity. Following implantation, a rigorous rehabilitation programme can be started, enabling early weight bearing and joint range of motion [18].

Additionally, because there are no sites for osteosynthesis, prosthetic reconstruction carries a lesser risk of deep infection than allografts, and non-union is not a problem. The longevity, complications, and functional outcome differ depending on the anatomic site, prosthesis type, and fixation method [19,20]. Our study's recommended modality for limb preservation is modular segmental-replacement system prosthesis. At 80 months, the prosthetic survival rate was 83.7%, while the total complication rate was 53.5%.

Numerous studies have been conducted to look at endoprosthetic survival rates following tumour resection. However, it is challenging to summarise the findings and conduct a systematic review due to the limited patient population and variety of endoprosthesis models and principles. The majority of tumour endoprosthetic survival rates range from 60% to 80% at five years and from 40% to 70% at 10 years [21,22]. The reported follow-up for the existing rotating-hinge knee design is just about 10 years. In 1995, Malawer et al. found that prostheses had a five-year survival rate of 83% and a 10-year survival rate of 67%. They had an 11% amputation rate, a 13% infection rate, a 15% revision rate, and a 6% local recurrence rate. In total, 44% of patients experienced at least one complication [23].

In our study, three participants (10.7%) had bone growth or deposition on the mega prosthesis' metal interface and bone, which will lessen the possibility that the prosthesis will become loose, dislocate, or fracture around the prosthesis. As a result, in the future, if we use hydroxyapatite-coated implants, fresh bone formation above the implant may provide additional stability. Although there was some extensor lag when using the anterior method, in our study we found no significant difference in results with any approach other than the anterior approach to the knee for tumour resection.

Limitations

So far, we have only seen three previous papers on this topic from India [24]. However, the retrospective methodology and the small number of patients in our study place certain limitations on it.

## Conclusions

In our study, the most common type of benign tumour of the bones was giant cell tumour including recurrence of giant cell tumour (GCT), so the Kaplan-Meier method analysis found that relapse-free survival was more than eighty percent among enrolled subjects during the median follow-up period. Additionally, in our study, bone growth or deposition was observed at the metal-to-bone interface of the mega prosthesis, reducing the risk of prosthesis fracture, dislocation, or loosening around the prosthesis. As a result, in the future, if we use hydroxyapatite-coated implants, fresh bone formation above the implant may provide additional stability. It can be difficult to surgically treat patients who have malignant bone tumours. In limb-sparing surgery for bone tumours, the modular segmental-replacement system prosthesis that we preferred, along with timely standard chemotherapy, produced satisfactory results in terms of tumour control and limb function. To get good long-term results, the case selection must be appropriate. Its fundamental drawback is the need for specialised infrastructure. In circumstances where limb preservation is not possible, amputation remains a viable option.
